# The perichromatin region of the plant cell nucleus is the area with the strongest co-localisation of snRNA and SR proteins

**DOI:** 10.1007/s00425-012-1640-z

**Published:** 2012-04-24

**Authors:** Janusz Niedojadło, Zbigniew Mikulski, Konrad Dełeńko, Adriana Szmidt-Jaworska, Dariusz J. Smoliński, Alan L. Epstein

**Affiliations:** 1 Department of Cell Biology, Institute of General and Molecular Biology, Nicolaus Copernicus University, Ul. Gagarina 9, 87-100 Torun, Poland; 2 Department of Plant Physiology and Biotechnology, Nicolaus Copernicus University, 87-100 Torun, Poland; 3 Department of Pathology, USC Keck School of Medicine, Los Angeles, CA USA; 4 Present Address: Division of Inflammation Biology, La Jolla Institute for Allergy and Immunology, La Jolla, CA 92037 USA

**Keywords:** Architecture of plant cell nucleus, PANA antigen, SnRNA, Splicing, SR proteins

## Abstract

The spatial organisation of the splicing system in plant cells containing either reticular (*Allium cepa*) or chromocentric (*Lupinus luteus*) nuclei was studied by immunolabelling of SR proteins, snRNA, and the PANA antigen, known markers for interchromatin granule clusters in mammalian cells. Electron microscope results allowed us to determine the distribution of these molecules within the structural domains of the nucleus. Similar to animal cells, in both plant species SR proteins were localised in interchromatin granules, but contrary to animal cells contained very small amounts of snRNA. The area with the strongest snRNA and SR protein co-localisation was the perichromatin region, which may be the location of pre-mRNA splicing in the plant cell nuclei. The only observable differences in the organisation of reticular and chromocentric nuclei were the size of the speckles and the number of snRNA pools in the condensed chromatin. We conclude that, despite remarkable changes in the nuclear architecture, the organisation of the splicing system is remarkably similar in both types of plant cell nuclei.

**Electronic supplementary material:**

The online version of this article (doi:10.1007/s00425-012-1640-z) contains supplementary material, which is available to authorized users.

## Introduction

Splicing is the process that is responsible for the removal of intron sequences from pre-mRNA and requires U1, U2, and U4/U5/U6 snRNPs (Krämer 1996), as well as SR proteins (Birney et al. 1993). This process is a fundamental molecular event that is regulated at several stages of gene expression in all Eukaryota. Despite the high degree of conservation in the splicing process among species, introns and spliceosome elements differ between plants and animals. Plant introns are shorter, contain more AU and are highly variable in their 5′ and 3′ splice sites, as well as in their branch point consensus sequences (Schuler 2008). Among the splicing factors in plants, there are a large number of homologues within individual categories of snRNAs and SR proteins. A genome-wide survey in *Arabidopsis* identified a total of 70 genes encoding snRNAs, most of which seem to be active as their promoter regions contain both TATA box and conserved upstream element (USE) motifs (Wang and Brendel 2004). In *Arabidopsis* and rice, 19 and 22 SR genes were found, respectively, and only 12 SR genes have been identified in the human genome (Barta et al. 2010). Among all the currently known SR plant proteins, there are at least four SR plant proteins that are specific to the plant kingdom (Barta et al. 2008).

At the electron microscopy level, it was first shown that Cajal bodies (CBs) are the sites of snRNPs in plant cells (Vázquez-Nin et al. 1992; Testillano et al. 1993; Gulemetova et al. 1998). Cajal bodies are a prominent and multifunctional structure in plant somatic and generative cells (Zienkiewicz and Niedojadło 2004; Lorković and Barta 2008). Similar to animal CBs, plant CBs participate in the transcription and processing of snRNAs (Schul et al. 1998; Boundonck et al. 1999; Darzacq et al. 2002). Recently, only CB functions that are specific to plant cells have been identified. For example, in plant cells, CBs participate in the biogenesis of siRNAs (Pontes and Pikaard 2008). Additionally, CBs in meiocytes may contain mRNA during certain developmental stages (Smoliński and Kołowerzo 2012).

The second structure involved in the organisation of the splicing system is the interchromatin network, which can be visualised by light microscopy using U2B antibodies or molecular probes specific for U1 and U2 snRNAs. The interchromatin network was described in *Pisum sativum* (Beven et al. 1995), *Saccharum officinarum* (Acevedo et al. 2002), *Arabidopsis thaliana* (Boundonck et al. 1998), and *Allium cepa* (Cui and Moreno Díaz de la Espina 2003), but its role in the functioning of the splicing system has not been determined to date.

The eukaryotic spliceosome contains SR proteins in addition to snRNAs*.* They are characterised by the presence of one or two RNA-binding domains of the RRM type, and a reversible phosphorylated arginine/serine-rich (RS) domain (Barta et al. 2008). Using fusion fluorescent proteins, SR proteins in plant cell nuclei were described, for the first time (Ali et al. 2003; Docquier et al. 2004; Fang et al. 2004), as speckles similar to those seen in animal cells. Plant speckles are morphologically diverse structures, and their shape and size depend on the species, cell type, and stage of development (Ali et al. 2003; Fang et al. 2004; Lorković et al. 2004). Treatment of plant cells with transcription and phosphorylation inhibitors results in the migration of SR proteins and the enlargement of speckles (Ali et al. 2003; Docquier et al. 2004; Fang et al. 2004). These results suggest that speckles in plants, similar to animal cell speckles, can function as storage sites and locations for SR protein assembly (Lamond and Spector 2003). In contrast to animals (Phair and Misteli 2000), the movement of SR proteins in *Arabidopsis* is ATP dependent (Ali and Reddy 2006). Additionally, the molecular composition of these structures is not well understood. These two factors inhibit our ability to determine if speckles in plant cells have the same role as in animal cells. Furthermore, our limited understanding of the functional organisation of the splicing system with regard to the spatial interactions of snRNAs and SR proteins also hinders our efforts to elucidate the functional role of these nuclear structures in plant cells.

In the present investigation, the localisation of snRNAs, SR proteins, and the PANA antigen was studied in two types of plant cell nuclei (chromocentric nuclei present in *Lupinus luteus* and reticular nuclei present in *Allium cepa).* The PANA antigen is a marker of interchromatin granules in animals. We expected that, similarly to animal cells, antibodies to the PANA antigen would more precisely label speckles and their counterpart interchromatin granules than reagents detecting SR proteins. Immunolabelling at the electron microscope level allowed us to determine which nuclear domains were enriched with these molecules. Utilising these methods enabled us to identify splicing regions in the plant cell nucleus as areas of strong co-localisation of snRNAs and SR proteins.

## Materials and methods

### Materials

Bulbs of *Allium cepa* L. (Horticulture Farm in Toruń, Poland) were placed on a wire mesh covering a container full of tap water so that only the root blastema was exposed to water. After 2–3 days, the cultured bulbs developed 1–2 cm roots. *Lupinus luteus* cv Zeus (Torseed SA Toruń, Poland) seeds were soaked in water for 5 h and subsequently germinated at 18 °C for 2 days on water-soaked tissue paper. Meristems of *Allium cepa* and *Lupinus luteus* roots were excised under water and fixed in 4 % paraformaldehyde in 50 mM Pipes buffer, pH 7.0 for 12 h at 4 °C. Fixed roots were washed three times for 15 min in Pipes buffer and 15 min in PBS buffer. Samples for electron microscopy were prepared by fixing roots in 4 % paraformaldehyde with 0.25–1 % glutaraldehyde in the Pipes buffer pH 7.0.

For immunoblotting, HeLa cells were grown in EMEM (Sigma-Aldrich, St. Louis, MO, USA) supplemented with 10 % FCS (Sigma-Aldrich), 1 % non-essential amino acids (Sigma-Aldrich), penicillin, and streptomycin at 37 °C in 5 % CO_2_.

### Immunofluorescence labelling

The fixed and washed roots were dehydrated in a series of increasing ethanol concentrations with 10 mM dithiothreitol and embedded in BMM resin (butyl methacrylate, methyl methacrylate, 0.5 % benzoin ethyl, 10 mM dithiothreitol; Fluka Chemie, Buchs, Switzerland). The embedded sample was cut into 2 μm sections, which were placed on Biobond-covered microscope slides. The BMM resin was dissolved in acetone. Slides were washed three times for 10 min in water, incubated for 15 min in PBS, blocked with 0.02 % acetylated BSA for 30 min, and incubated with primary antibody diluted in 0.01 % acetylated BSA in PBS for 16 h at 4 °C. SR proteins were detected by the 3C5 mouse IgM antibody (a gift from D.L. Spector Cold Spring Harbor Laboratory, NY, USA) (diluted 1:100) and antigen PANA by the mouse IgM 780-3 antibody (diluted 1:25) (Clevenger and Epstein 1984). Slides were washed in PBS and incubated with secondary antibody diluted in PBS buffer. Goat anti-mouse IgM antibody labelled with Alexa Fluor 488 (Molecular Probes, NY, USA) and goat anti-mouse IgM antibody labelled with Cy3 (Sigma-Aldrich) were used at a 1:100 dilution. Control reactions were performed without the primary antibodies. DNA was stained with 4,6-diamidino-2-phenylindole (DAPI) (Sigma-Aldrich) following the manufacturer’s instructions. The immunolocalisation results were analysed with a Nikon A1 confocal microscope with 0.3-μm step interval.

### Fluorescence in situ hybridisation

The U2 snRNA was detected using the following oligonucleotides: 5′-ATATTAAACTGATAAGAACAGATACTACACTTG-3′. The probe was labelled chemically with Cy3 fluorochrome at the 5′ end (IBB PAN, Warsaw, Poland). After a 1-h prehybridisation, the hybridisation was performed for at least 12 h at 37 °C in the hybridisation buffer (30 % formamide, 4× SSC, 100 μg/ml of herring sperm DNA). The slices were then washed in 4× SSC, 2× SSC, and 1× SSC. DNA was stained with DAPI (Sigma-Aldrich). The control reaction was performed in the same way using the hybridisation buffer without a probe.

### Immunogold labelling

After fixation, roots were dehydrated in ethanol, then infiltrated with and embedded in the LR White (Electron Microscopy Sciences, Fort Washington, PA, USA) or LR Gold (Sigma-Aldrich) resin. The 100-nm sections were sliced using a Leica Ultracut UCT ultramicrotome and mounted on formvar-coated nickel grids. The grids with sections were treated with 1 % BSA and incubated for 17 h at 4 °C with primary antibody or, for double labelling experiments, with a mixture of primary antibodies diluted in PBS with 1 % BSA (Sigma-Aldrich). The primary antibodies were used in the following dilutions: 3C5 (1:10), 780-3 (1:30) and anti-3mG antibodies (Calbiochem, San Diego, CA, USA) (1:30–1:100). Colloidal gold-conjugated secondary antibody was diluted in PBS with 1 % BSA and reacted with sections for 45 min at 35 °C. Finally, the sections were stained with 2.5 % uranyl acetate (20 min) and, for some samples, with 2.5 % lead citrate and examined using TEM (Joel 1010) at 80 kV. In control specimens, the sections were treated with the reaction mixture and primary antibodies were omitted.

### Immunoblotting

The roots of onion and lupine were frozen in liquid nitrogen and homogenised using a mortar and pestle. Next, the nuclei were isolated using the CelLytic PN extraction kit from Sigma-Aldrich with the semi-pure preparation option and following the manufacturer’s instructions. HeLa cells were sonicated three times for 10 s. The homogenate was suspended in extraction buffer (20 mM Tris–HCl, pH 7.5, 2.5 µM EDTA, 5 mM NaF, 10 µM aprotinin, 10 µM leupeptin and 1 µM PMSF) and incubated on ice for 15 min. The crude protein extracts were centrifuged at 16,000*g* at 4 °C for 30 min. The pellet concentration was determined by the Bradford method (1976) using BSA as the standard.

Aliquots of soluble protein extracts from onion and lupine root nuclei (60 μg) were incubated in protein sample buffer at 95 °C for 10 min and resolved using 10 % (w/v) SDS-PAGE as described by Laemmli (1970). Proteins were transferred to PVDF membrane by a semi-dry system (BioRad, Hercules, CA, USA) (15 min at 15 mA) using 25 mM Tris, 192 mM glycine, and 20 % (v/v) methanol (pH 8.3). Membranes were blocked in PBS containing 3 % non-fat dry milk and then incubated overnight at 4 °C with a primary monoclonal antibody against PANA. After washing three times in PBS, the membranes were incubated for 1–2 h with secondary horseradish peroxidase conjugated to goat anti-mouse IgM antibody diluted 1:100,000 in PBS buffer and bands were visualised by chemiluminescence using the ECL plus system (GH Healthcare).

### Quantitative evaluation of signal

For fluorescence signal evaluation, 20–35 nuclei from three different preparations were analysed. The results were registered using a Nikon A1 confocal microscope. The three-dimensional optical sections were acquired with a 0.3-µm step interval. For all antigens, the obtained data were corrected for background autofluorescence determined by negative control signal intensities. The analysis was performed using NIS-Elements AR3.00 (Nikon, Laboratory Imaging). The data in the figures and text are expressed as the mean ± SD. The non-parametric rank based Kruskal–Wallis test was used to compare multiple groups, and if significant differences were detected, the Mann–Whitney test was then used to compare the two experimental groups. The tests were performed using GraphPad Prism 5 software (GraphPad, La Jolla, CA, USA). *P* ≤ 0.05 was considered significant, and a *P* ≤ 0.01 was considered highly significant.

For immunogold signal evaluation, ten images (each containing one nucleus) for each antigen were taken for gold grains analysis. The signal density was evaluated in the following six nuclear compartments: the perichromatin region, condensed chromatin, interchromatin granule clusters, interchromatin space (excluding the interchromatin granule clusters), nucleolus, Cajal bodies, and cytoplasm. The perichromatin region was delineated as the periphery of condensed chromatin areas, which represents the interface between condensed chromatin and the interchromatin space, and was arbitrarily defined as a band 80 nm in width (Niedojadło et al. 2011). Each compartment was determined and its area was measured using the NIS-Elements AR3.00 software (Nikon, Laboratory Imaging). The number of gold particles was counted, and the labelling density for each nuclear compartment was expressed as the number of gold particles per μm^2^. Statistical analysis was conducted as described above.

## Results

### Localisation of SR proteins, snRNP, and PANA antigen in reticular and chromocentric types of plant cell nuclei

**Fig. 1:**
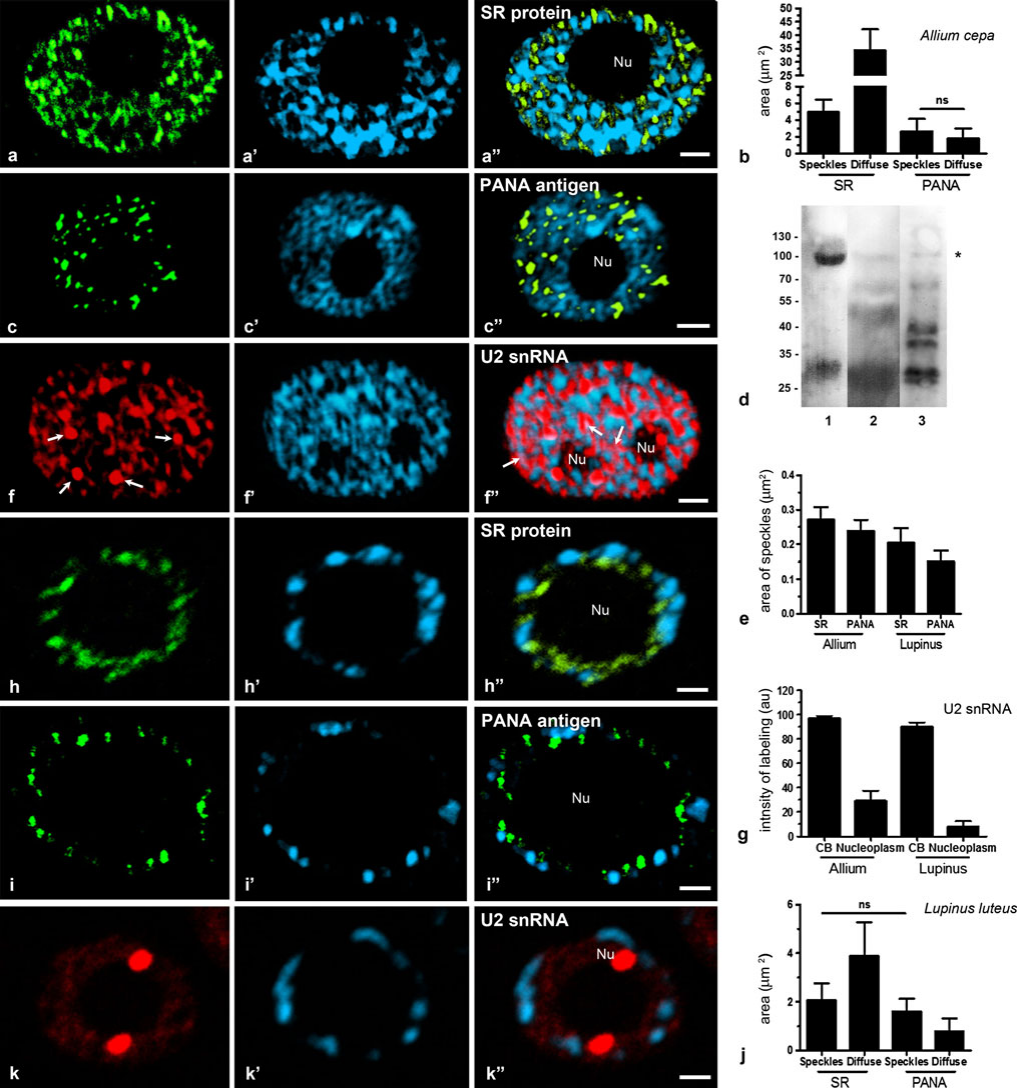
Localisation of SR proteins, PANA antigen and U2 snRNA in reticular (*Allium cepa* **a**, **c**, **f**) and chromocentric nuclei (*Lupinus luteus* **h**, **i**, **k**). **a** Localisation of SR proteins in the meristematic cells of roots. **b** The total area of nuclear structures similar to speckles and diffuse region stained with antibodies to SR proteins and to the PANA antigen in the *Allium cepa* nuclei. *Ends of lines* (*ns*) indicate regions of nucleus with nonsignificant difference. **c** Localisation of PANA antigen. There are structures resembling speckles in the nucleus of *A*. *cepa.* **d** Western blot of PANA antibody with total protein extracts from HeLa cells (*1*), nuclei of roots *Allium cepa* (*2*), and nuclei of roots *Lupinus luteus* (*3*). **e** The size of nuclear structures stained with antibodies to SR proteins and to the PANA antigen in the *A. cepa* and *L. luteus* nuclei. **f** Localisation U2 snRNA. The bright spots are Cajal bodies (*arrows*). Diffuse staining of the nucleoplasm is also observed. **f**″ Co-localisation of signals from U2 snRNA and DNA staining with DAPI were observed (*arrows pink in colour*). **g** The intensity of labelling in *CB* and nucleoplasm stained with U2 probe. Nuclear localisation of SR proteins (**h**) and PANA antigen (**i**) in a chromocentric nucleus. **j** The intensity of labelling of speckles and diffuse nuclear fraction stained with antibodies to SR proteins and to the PANA antigen of *L. luteus*. *Ends of lines* (*ns*) indicate regions of nucleus with nonsignificant difference. **k** There are two Cajal bodies and a diffuse pool stained U2 probe in the nucleus of *L. luteus*. **a**′, **c**′, **f**′, **h**′, **i**′, **k**′ DAPI staining. **a**″,**b**″,**f**″,**h**″,**i**″,**k**″ Overlay DAPI staining and corresponding antigens. *Nu* nucleolus. *Bar* 5 μm

In onion meristematic cells, SR proteins were detected using 3C5 antibodies and confocal microscopy. These antibodies were previously used in mono- and dicotyledonous plants (Fang et al. 2004; Zienkiewicz et al. 2006). Immunoreactivity was present in the form of distinct spots similar to speckles and as a diffuse pool in the extra-nucleolar nucleoplasm of the cell nucleus (Fig. 1a). The total area occupied by speckles was seven times lower than the diffuse form (Fig. 1b). The intensity of labelling in the speckles was about two-and-half times more than in the diffuse area (Supplemental data Fig. S1a). Projections of confocal sections from nucleus after 3C5 immunolabelling showed speckles as separate structures of different size (Supplemental data Fig. S1b). An interesting pattern of SR protein localisation was observed in the nuclei of root epidermal cells, which appear to be more intensely stained than meristematic cell nuclei (Supplemental data Fig. S1c). Controls performed by omitting the incubation with the 3C5 antibody showed no staining in the nucleus (Supplemental data Fig. S3a).

To better define speckles, we used the 780-3 monoclonal antibody that recognises the PANA antigen, which was previously found to be a marker of interchromatin granules in animal cells (Clevenger and Epstein 1984). In onion root meristematic cells, the PANA antigen was seen as speckles, which occasionally formed elongated shapes (Fig. 1c) and were mostly found in regions of the nucleus that were weakly stained with DAPI (Fig. 1c″). Additionally, in epidermal cells the PANA antigen labelled nuclear structures with a high signal concentration, which was similar to the SR protein staining pattern (Supplemental data Fig. S1d). Control treatments consisting of incubations without the primary antibody showed very little or no signal (Supplemental data Fig. S3b). Characterisation of the antibodies by Western blot confirmed two bands of approximately 105 and 30 kDa in HeLa cells, as first identified by Clevenger and Epstein (1984) (Fig. 1d). In isolated plant nuclei, 780-3 recognised four additional bands, 66-, 39-, 37- and 27-kDa bands, in lupine, and two additional bands, 66- and 49-kDa bands, in onion.

The area of individual speckles stained by antibodies for the SR proteins was 13 % larger than the area stained by the PANA antigen in the meristematic cells of *Allium cepa* roots (Fig. 1e).

The second group of splicing factors consists of snRNAs. To compare the pattern of the localisation of snRNAs and antigens forming the speckles*,* in situ hybridisation of U2 snRNA was conducted. In situ hybridisation of U2 snRNA showed signals in large clusters representing Cajal bodies (CBs) and in diffuse pools filling the extra-nucleolar part of the nucleoplasm (Fig. 1f). The intensity of labelling in the CBs was more than three times higher compared to the diffuse nucleoplasm (Fig. 1g). The diffuse pools were often found in the interchromatin space, but we also observed a co-localisation of signals from U2 snRNA and DNA staining with DAPI (Fig. 1f″, pink colour).

Parallel studies were conducted in lupine root cells and showed that the localisation patterns of SR proteins (Fig. 1h) and the PANA antigen (Fig. 1i) were similar to the patterns observed in onion cells. It appears that the SR protein speckles merge with each other, making them less distinctive when compared with analogous structures in the onion. The total area of the speckles labelled with the 3C5 antibody was 17 % larger than the area labelled with the 780-3 antibody (Fig. 1e). Projections of confocal sections after 3C5 immunolabelling showed few speckles in the nucleus of meristematic cells (Supplemental data Fig. S1e). The area of the speckles detected with the 3C5 antibody was almost half the area of diffuse labelling. In contrast, the area occupied by speckles stained with antibodies for the PANA antigen was two times larger than the diffuse area (Fig. 1j). The intensity of labelling in the speckles was about three times higher than in the diffuse area (Supplemental data Fig. S1a). Additionally, lupine root epidermal cells were stained more strongly with anti-SR antibodies than meristematic cells (Supplemental data Fig. S1f). Controls performed by omitting incubation with the primary antibody showed no staining in the nucleus (Supplemental data Fig. S3d, e).

Next, we examined the distribution of the U2 snRNA by in situ hybridisation. Labelling was localised to prominent spherical structures, corresponding to coiled bodies and to a diffuse pool (Fig. 1k). The level of U2 snRNA was about nine times higher than in the nucleoplasm (Fig. 1g). The label was excluded from the nucleolus and condensed chromatin. For in situ hybridisations and immunofluorescence methods, control treatments consisting of incubations without the probes or primary antibody were performed and showed no unspecific labelling (Supplemental data Fig. S3f).

Difference in the size of SR protein and PANA speckles in meristematic cells was also observed in the both species. Speckles stained with the 3C5 antibody were 11 % smaller in lupine cells than in onion cells (*P* ≤ 0.05, Fig. 1e). Interestingly, in both of the studied species, speckles labelled with the 3C5 antibody were larger than speckles stained with the 780-3 antibody.

These results indicate that in plant cell nuclei, antibodies against SR proteins and the PANA antigen are found in spots of various sizes, similar to speckles. A comparison of the distribution of SR proteins and PANA antigen with snRNP components indicates that areas of their highest concentration may represent different regions of the cell nucleus in both reticular and chromocentric types of cells.

### Identification of nuclear domains containing SR proteins, snRNAs, and the PANA antigen

**Fig. 2:**
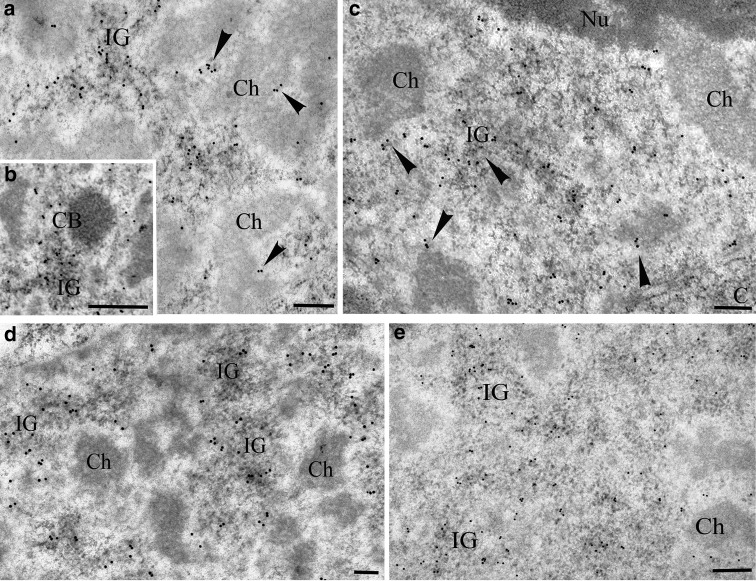
Immunogold localisation of SR proteins and PANA antigen. **a** Gold particles reveal that SR proteins mostly occurred in interchromatin granules (*IG*), as well as in the perichromatin region (*arrowheads*) and the interchromatin space of *A. cepa* nuclei. **b** Condensed chromatin (*Ch*) and Cajal bodies (*CB*) are devoid of gold grains. **c** A similar SR protein distribution pattern was observed in *L. luteus*. **d**, **e** After staining with anti-PANA antigen, gold particles were mainly observed in the interchromatin granules of *Allium cepa* (**d**) and *Lupinus luteus* (**e**). *PCh* perichromatin area, *IG* interchromatin granules, *Ch* condensed chromatin, *Nu* nucleolus, *CB* Cajal body. *Bar* 200 nm

**Fig. 3:**
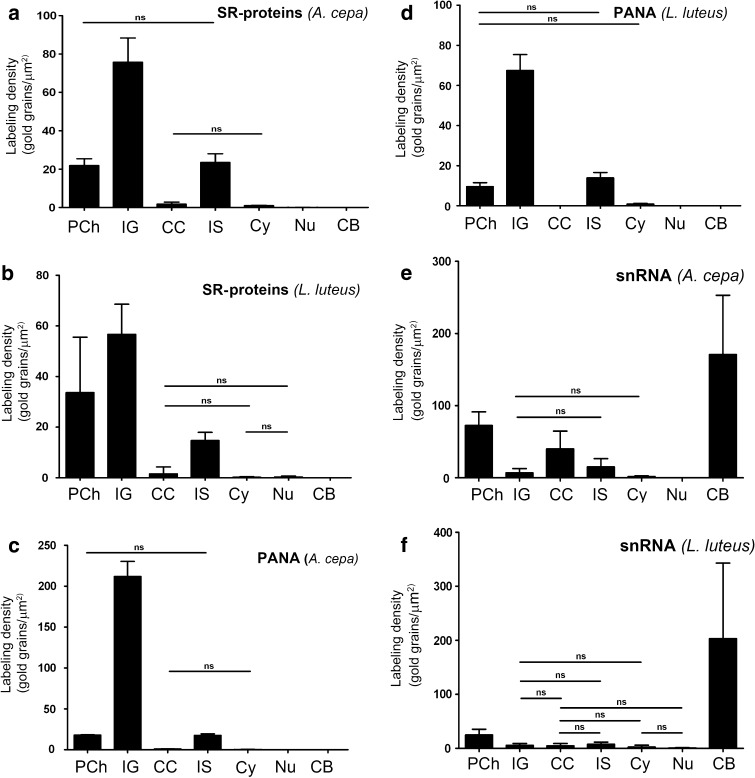
Quantitative analysis of immunogold localisation of anti-SR proteins, anti-PANA antigens, and anti-2,2,7 trimethylguanosine snRNA in the *A. cepa* and *L. luteus* nuclear domains. **a** *A. cepa* SR proteins. **b** *L. luteus* SR proteins. **c** *A. cepa* PANA antigens. **d** *L. luteus* PANA antigens. **e** *A. cepa* snRNAs. **f** *L. luteus* snRNAs. *ns* nonsignificant. *Ends of lines* (*ns*) indicate regions of nucleus with nonsignificant differences. *PCh* perichromatin area, *IG* interchromatin granules, *CC* condensed chromatin, *IS* interchromatin space, *Cy* cytoplasm, *Nu* nucleolus, *CB* Cajal body

At the electron microscope level, SR proteins in onion cell nuclei are most abundant in interchromatin granules (Fig. 2a), which in plant nuclei do not form typical clusters as in animals, but instead fill a significant part of the interchromatin space. Labelling intensity and distribution of interchromatin granules indicate that they represent the speckles observed at the light microscope level. A smaller, but significant, amount of the signal was localised along the border of condensed chromatin, i.e. in the perichromatin area and in the interchromatin space (which is defined as the interchromatin area excluding the perichromatin area, interchromatin granules, and Cajal bodies). In the perichromatin area, grains of gold occasionally formed clusters similar to clusters in the interchromatin granules (Fig. 2a). No signal or only individual grains of gold were observed in Cajal bodies (Fig. 2b) and in condensed chromatin (Fig. 2a, b). A statistical analysis of the amount of gold grains per μm^2^ in morphologically specified areas of the nucleus showed that there was no difference in the signal level between the perichromatin area and the interchromatin space (Fig. 3a). Distribution of SR proteins in the perichromatin and interchromatin space suggests that these areas represent the diffuse pool observed at the confocal microscopy level. Control reaction after incubation without primary antibody confirmed the specificity of the obtained the signal. (Supplemental data Fig. S3g).

In comparison with meristematic cells, onion root epidermal cells stained with uranyl acetate and lead citrate showed that the interchromatin space was usually strongly stained, which suggests that there was a higher concentration of proteins and RNA in the interchromatin space. Furthermore, interchromatin granules were more frequently seen as clusters, and perichromatin fibrils were more distinguishable from chromatin and other interchromatin areas (compare Fig. 2a with Supplemental data Fig. S1c). Despite these ultrastructural differences, the SR protein distribution pattern was very similar to that observed in meristematic cells (compare Fig. 2a with Supplemental data Fig. S1a).

In nuclei of lupine root cells, observations and statistical analysis show similar patterns in SR protein distribution as those found in onion (Figs. 2c, 3b). However, in a nucleus of lupine, SR proteins were more abundant in the perichromatin area than in the interchromatin space (Fig. 3b).

Localisation of the PANA antigen at the electron microscope level revealed strong labelling of interchromatin granules in onion (Fig. 2d) and lupine (Fig. 2e) cells. In onion cells, the amount of the PANA antigen in interchromatin granules was over 11 times higher than in the perichromatin area and interchromatin space (Fig. 3c). In lupine cells, the amount of the PANA antigen in interchromatin granules was almost 7.5 times higher than that in the perichromatin area and 4.8 times higher than that in the interchromatin space (Fig. 3d). These data imply that in reticular and chromocentric nuclei, the PANA antigen is mainly found in interchromatin granules and that other regions of the nucleus only contain a small amount of the PANA antigen. Control for *L. luteus* and *A. cepa* in which the primary antibodies were omitted resulted in a lack of labelling (Supplemental data Fig. S3g, h).

**Fig. 4:**
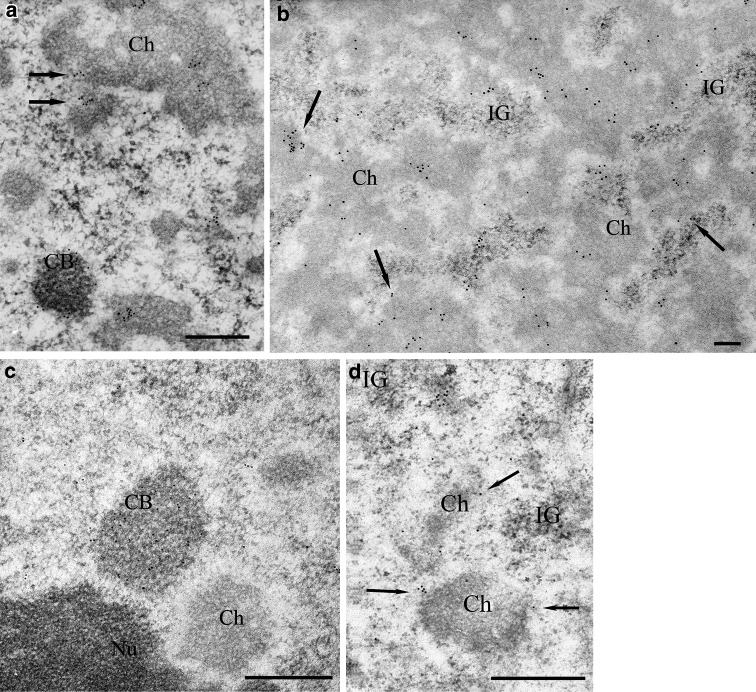
Localisation of snRNAs in reticular and chromocentric plant nuclei. **a** The structures with the strongest labelling in the *Allium* nucleus were Cajal bodies (*CB*). Grains of gold were also found in the perichromatin area (*arrows*) and in the dense chromatin (*Ch*). **a**, **b** Gold grains were rarely observed in the interchromatin granules (*IG*). **c** *Lupinus* Cajal bodies (*CB*) and the perichromatin area (*arrows*) are labelled. **c**, **d** In contrast to the *Allium* nucleus, labelling was not found in the condensed chromatin. *Bar* 200 nm

U2 snRNA was detected by in situ hybridisation. Although the overall signal intensity was weak, we were able to detect U2 snRNA which was localised at the chromatin periphery and in the condensed chromatin (Supplemental data Fig. S2b, c). Control reactions after incubation without the primary antibody confirmed the specificity of the signal obtained (Supplemental data Fig. S3i). To verify snRNAs detection, immunogold localisation for snRNA was also conducted. Small nuclear RNA was detected using an antibody (3mG) against 2,2,7 trimethylguanosine snRNAs, which showed strong labelling in the Cajal bodies of both species (Fig. 4a, c). In onion cells, a large amount of the signal was also found in the perichromatin area. Grains of gold were observed in condensed chromatin. In that compartment, the signal was observed as clusters and individual gold grains (Fig. 4a, b). In the interchromatin space, the signal was almost five times lower than in the perichromatin area and over 2.5 times lower than in the condensed chromatin (Fig. 3e). The structures in the nuclei with the lowest amount of signal, apart from the nucleolus, were the interchromatin granules (Figs. 3e, 4a, b). The specificity of reaction was controlled by treating sections with RNase, DNase, and K proteinase before incubating them in the antibody against 3mG. In the RNase-treated samples, individual grains of gold were localised and did not accumulate in the chromatin or perichromatin. Unfortunately, RNase digestion may result in considerable deterioration of nuclear ultrastructures, which would make the identification of interchromatin domains difficult (Supplemental data Fig. S2d). Digesting with DNase and K proteinase resulted in a slight decrease in the signal and did not affect the snRNAs distribution pattern (data not published). To verify snRNAs detection, in situ hybridisation for U2 snRNA was also conducted. In lupine samples, similar to onion samples, large quantities of gold grains were observed in the perichromatin area and in CBs, which indicated the presence of 3mG (Fig. 4c, d). Only individual grains of gold were found in the condensed chromatin. A very weak signal was seen in interchromatin granules (Fig. 4d).

**Fig. 5:**
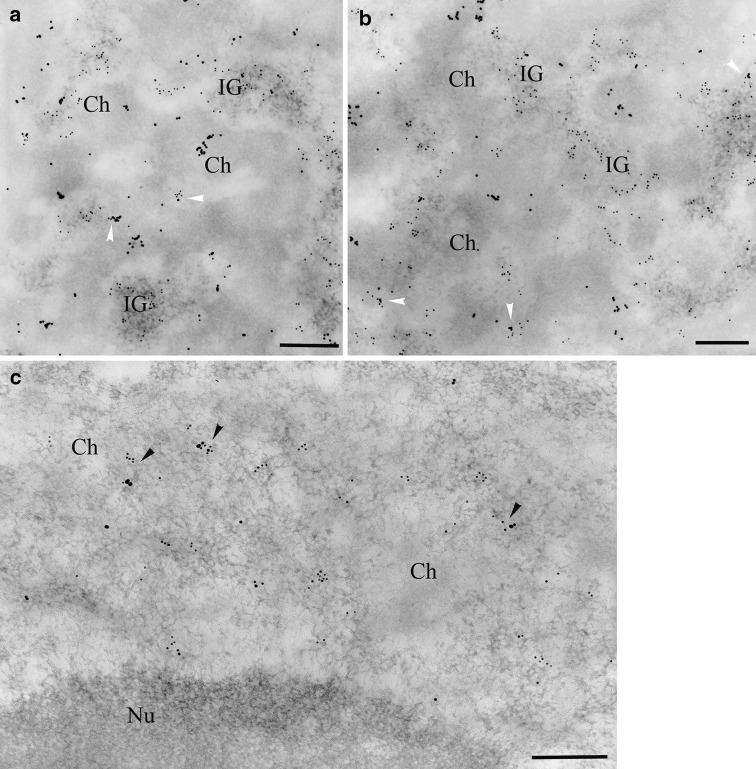
Simultaneous double labelling of SR proteins and snRNAs in *Allium cepa* (**a**, **b**) and *Lupinus luteus* (**c**). SR proteins were detected by 10-nm colloidal gold particles and were mainly distributed in the interchromatin granules, whereas snRNAs, which was visualised by 15-nm gold grains, was found in the perichromatin and condensed chromatin areas. The strongest co-localisation of both antigens was observed in the perichromatin area (*arrows*). *IG* interchromatin granules, *Ch* condensed chromatin, *Nu* nucleolus. *Bar* 200 nm

Co-localisation for snRNAs and SR proteins was also performed. Although the labelling was generally weaker, the distribution pattern of the signal was consistent with the data from individual localisation experiments (Fig. 5a, b, c). Additionally, our results show that the strongest co-localisation of snRNAs and SR proteins occurs in the perichromatin area of both species.

## Discussion

In this study, we provide evidence that the organisation of the splicing system is similar in reticular and chromocentric plant cell nuclei despite the remarkable differences in their nuclear architecture. Additionally, our results show a low level of co-localisation of molecules forming the spliceosome, i.e. snRNAs and SR proteins in the nuclei of both species. Using immunogold electron microscopy, we identified the domains of the nucleus that are enriched with each of these antigens.

Our snRNP localisation results observed at the light microscope level confirm the presence of the interchromatin network (Beven et al. 1995; Cui and Moreno Díaz de la Espina 2003). However, our studies at the electron microscope level showed that in both types of nuclei, snRNAs were found in the perichromatin area in addition to CBs. These findings suggest that the interchromatin network observed at the light microscopic level corresponds to the perichromatin areas. In the model CT–IC (chromosome territory–interchromatin compartment) (Misteli 2007; Cremer and Cremer 2010), the perichromatin area connects the chromosome territories with an interchromatin compartment and could represent intermingling of chromosomal regions (Cremer and Cremer 2010). The presence of a large pool of snRNAs along the chromatin periphery is well known and has been described for animal cells. This snRNAs pool may be involved in the co-transcription splicing that occurs in the perichromatin area of the nucleus (Cmarko et al. 1999; Fakan 2004), where actively transcribed DNA is present (Niedojadło et al. 2011).

In the reticular type of nuclei, a significant amount of snRNAs was found in condensed chromatin. We observed this localisation pattern using an antibody against 3mG and a probe for U2 snRNA. These results were also verified in several control experiments. The additional presence of snRNAs in the condensed chromatin raises questions of its function in this region. The presence of snRNAs in condensed chromatin in the reticular nuclei of *Hyacinthus orientalis* L. pollen grains was previously shown by Zienkiewicz et al. (2008). The snRNAs were found in the chromatin of nuclei in which the transcriptional activity had been significantly reduced or inhibited, and may simply be a storage area for these molecules. This finding is supported by the lack of SR proteins, which are required for functional spliceosome, in this region of the nucleus. In animal cells, interchromatin granules are known storage domains for splicing factors (Lamond and Spector 2003). However, our studies at the ultrastructural level showed that in the interchromatin granules of onion and lupine meristematic cells, only a negligible amount of snRNAs was present. Therefore, we conclude that in reticular nuclei the condensed chromatin is most likely the storing domain for snRNAs.

In both types of cell nuclei, the degree of snRNAs and SR protein co-localisation observed at the light microscope level was low. Unlike snRNAs, SR proteins formed numerous speckles. Studies at the ultrastructural level showed that SR proteins, unlike snRNAs, were mainly localised in the interchromatin granules of both onion and lupine nuclei. These results are consistent with earlier studies by Fang et al. (2004), who suggested that the interchromatin granules discovered in plant cells could be the speckles observed at the light microscope level. According to studies in animal cells, interchromatin granules are storage sites for SR proteins that do not participate in splicing (Lamond and Spector 2003; Cremer et al. 2004). Our results, however, showed that a significant amount of SR proteins of the total SR protein pool in onion (40 %) and in lupine (53 %) nuclei was found outside interchromatin granules. This finding suggests that antibodies against SR proteins may not be good markers for splicing speckles. Additionally, the amount of SR proteins in onion and in lupine nuclei (approximately 30 and 60 %, respectively) observed in the interchromatin granules was found in the perichromatin area. In the perichromatin area, gold particles were occasionally found in clusters that formed a pattern similar to the pattern observed in interchromatin granules. These results combined with our light microscopy results suggest that the observed clusters of SR proteins may represent both the interchromatin granules and the perichromatin area. Similar findings on SR protein cluster patterns have also been found in the transcription region of animal cells (Misteli et al. 1997). The co-localisation of transcription regions and SR proteins was reported after applying large dilutions of antibodies (Neugebauer and Roth 1997). Therefore, these findings suggest that in plant cells, similar to animal cells, antibodies for SR proteins are not precise markers for speckles, which at the electron microscope level correspond to interchromatin granules.

An alternative marker that may label speckles more accurately and help distinguish nuclear domains at the light microscope level is the 780-3 antibody, which detects the PANA antigen. The 780-3 antibody, developed by Clevenger and Epstein in 1984, was the first antibody that could specifically identify interchromatin granules in animal cells. Our results show that this antibody recognises the same nuclear domain in plants. The amount of PANA antigen in interchromatin granules is 12 and 5 times higher in onion and lupine cell nuclei, respectively, than in the other labelled structures. This finding demonstrates that the PANA antigen is a better marker for interchromatin granules than SR proteins in plant cells. Western blot analysis of plant tissue (data not shown) and isolated nuclei extracts reveals that the 780-3 antibody detects another band (in addition to the 105- and 30-kDa bands), which was not identified in animal cells (Clevenger and Epstein 1984). This additional band in plants could represent a proteolytic fragment of the 105-kDa band. Alternatively, as suggested Clevenger and Epstein (1984), this band may represent monomeric forms of the 105-kDa antigen. We are unable to perform an immunoprecipitation analysis, as it had already been determined that this antibody cannot give positive results (Clevenger and Epsteins 1984). Despite the need for further biochemical studies of the PANA antigen, our electron microscope results indicate that the 780-3 antibody is a uniquely sensitive probe for interchromatin granules in plants. This antibody stained that specific nuclear domain in plants, and it may be a useful marker for studying the spatial organisation of the nucleus in plants, especially at the light microscope level.

With respect to the co-localisation of SR protein and snRNAs, the splicing region in plant cell nuclei is of great importance. Our statistical analyses of the individual antigens and our snRNAs and SR protein co-localisation study show that the region of the strongest co-localisation is the perichromatin area. A much lower degree of co-localisation is found in the interchromatin area. Taken together, these data suggest that in plants, similar to animals (Fakan 2004; Rino and Carmo-Fonseca 2009), splicing occurs along the periphery of condensed chromatin in both types of plant nuclei studied. Our results are partly consistent with recent studies on the co-localisation of some types of snRNAs and SR proteins, which were conducted at the light microscope level. Lorković et al. (2004) showed a low level of co-localisation between various types of snRNP and SR proteins in the mesophyll protoplasts of *Arabidopsis*. There is, however, a level of U1-70K and SRp34/SR1 co-localisation observed in structures similar to speckles. Additionally, Ali et al. (2008) using FRET and BiFC techniques in *Arabidopsis* showed that there was a level of U1-70K and SR45 co-localisation in speckles. These results suggest that while some specific snRNA and SR protein molecules are co-localised, the total level of snRNAs and SR protein co-localisation is low. Both of these studies were conducted on freshly isolated mesophyll protoplasts. Recently, it was shown that the organisation of chromatin and the nucleolus changes in freshly isolated protoplasts (Williams et al. 2003; Grafi 2004; Tessadori et al. 2007). Our electron microscopy results specifically show that there is no snRNAs and SR proteins co-localisation in the interchromatin granules of either onion or lupine root meristematic cells. The only areas in which these molecules are found simultaneously are the perichromatin areas, indicating that these areas are the only possible regions of splicing in the plant cell nuclei.

Our results also allow us to begin a functional comparison between reticular and chromocentric types of nuclei. We show that the splicing systems are similarly organised and that molecules related to splicing are similarly localised in the perichromatin areas of these two types of nuclei. Some differences, however, were also observed and include: (1) different sizes of speckles labelled with antibodies for the PANA antigen and SR proteins, which may be a result of the difference in spatial organisation of the interchromatin space of the species studied; (2) a significant difference in the amount of snRNAs on chromatin, which we believe to be a stored pool preferentially seen in reticular nuclei; and (3) differences in the ratios of the amounts of molecules found in the different nuclear domains. For all of the molecules studied, the differences in the amounts found between interchromatin granules and the perichromatin areas were significantly larger in onion nuclei than in lupine nuclei. Speckles labelled with the 3C5 antibody are much more visible in the onion nuclei than in lupine nuclei. Similar results were also obtained by Lorković et al. (2004) who used light microscopy to study the localisation of SR proteins in *Nicotiana*, which represented the reticular nucleus, and in *Arabidopsis*, representing the chromocentric nucleus. The speckles in tobacco nuclei were larger and more easily distinguishable than the diffuse pool observed in *Arabidopsis* nuclei. However, not in all nuclear domains, differences in the antigen amount related to splicing are larger in the reticular nuclei. A comparison of the amount of immunogold that labelled SR proteins between the perichromatin areas and the interchromatin spaces showed larger differences in lupine nuclei than in onion nuclei. In conclusion, the results presented above may be of significance in the selection of a model for experiments concerning the functional architecture of the cell nucleus in plants.

## Electronic supplementary material

Below is the link to the electronic supplementary material.

Supplementary material 1 (DOCX 10.7 kb)

Supplementary material 2 (JPEG 2.46 mb)

Supplementary material 3 (JPEG 7.21 mb)

Supplementary material 4 (JPEG 2.41 mb)

## Abbreviations

CB: Cajal bodies
DAPI: 4,6-Diamidino-2-phenylindole
PANA: Proliferation-associated nuclear antigen
SnRNP: Small nuclear ribonucleoproteins
SR: Serine/arginine-rich proteins
