# Supplementary material for: The perichromatin region of the plant cell nucleus is the area with the strongest co-localisation of snRNA and SR proteins
Source: Planta. 2012 Apr 24;236(2):715–26. doi: 10.1007/s00425-012-1640-z (PMC3404291; doi:10.1007/s00425-012-1640-z)
Supplement: Supplementary file 1 — Supplementary material 1 (DOCX 10.7 kb) [file 425_2012_1640_MOESM1_ESM.docx]

**Supplemental experimental procedures**

Hybridisation *in situ* at the EM level

Synthetic RNA probes consisting of 20-mer oligonucleotides for detecting U2 snRNA were labelled by the A plus tailing kit (Epicentre Biotechnologies) with digoxigenin-UTP (Roche). Grids with sections were incubated in hybridisation buffer containing: 100-200 pmol/ml probe, 4xSSC, 1 mg/ml DNA from herring sperm (Sigma-Aldrich), 10% dextran sulphate, and 30% formamide. Hybridisation was performed at 42°C in a humidified chamber overnight. The signal was visualised by incubation for 1 h in sheep anti-digoxigenin antibody conjugated to 10 nm gold particles (BioCell). In control experiments the probe was omitted from the hybridisation buffer.
