# Supplementary figures and images for: The perichromatin region of the plant cell nucleus is the area with the strongest co-localisation of snRNA and SR proteins
Source: Planta. 2012 Apr 24;236(2):715–26. doi: 10.1007/s00425-012-1640-z (PMC3404291; doi:10.1007/s00425-012-1640-z)

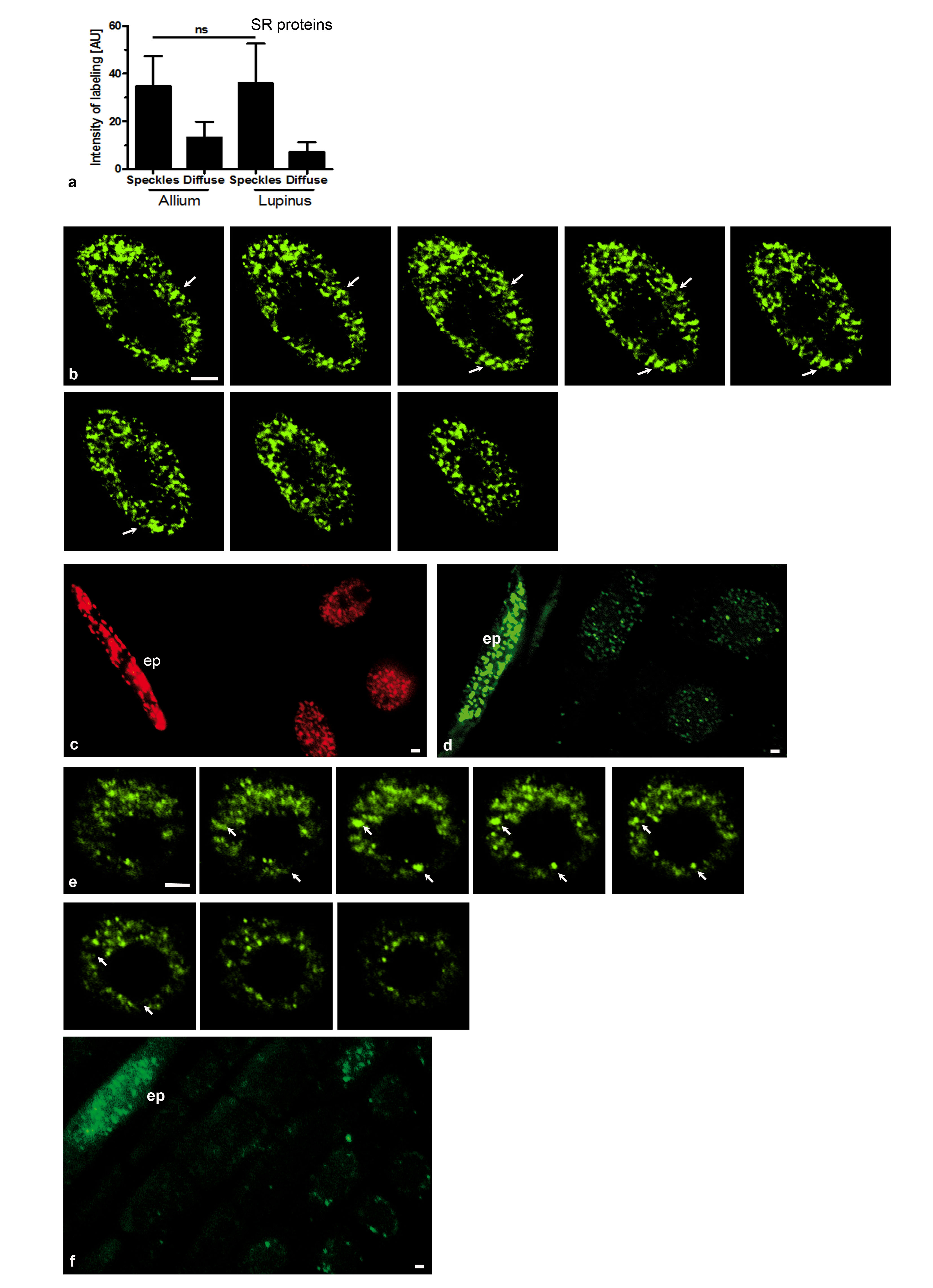

Supplement: Supplementary file 2 — Supplementary material 2 (JPEG 2.46 mb) [file 425_2012_1640_MOESM2_ESM.jpg]

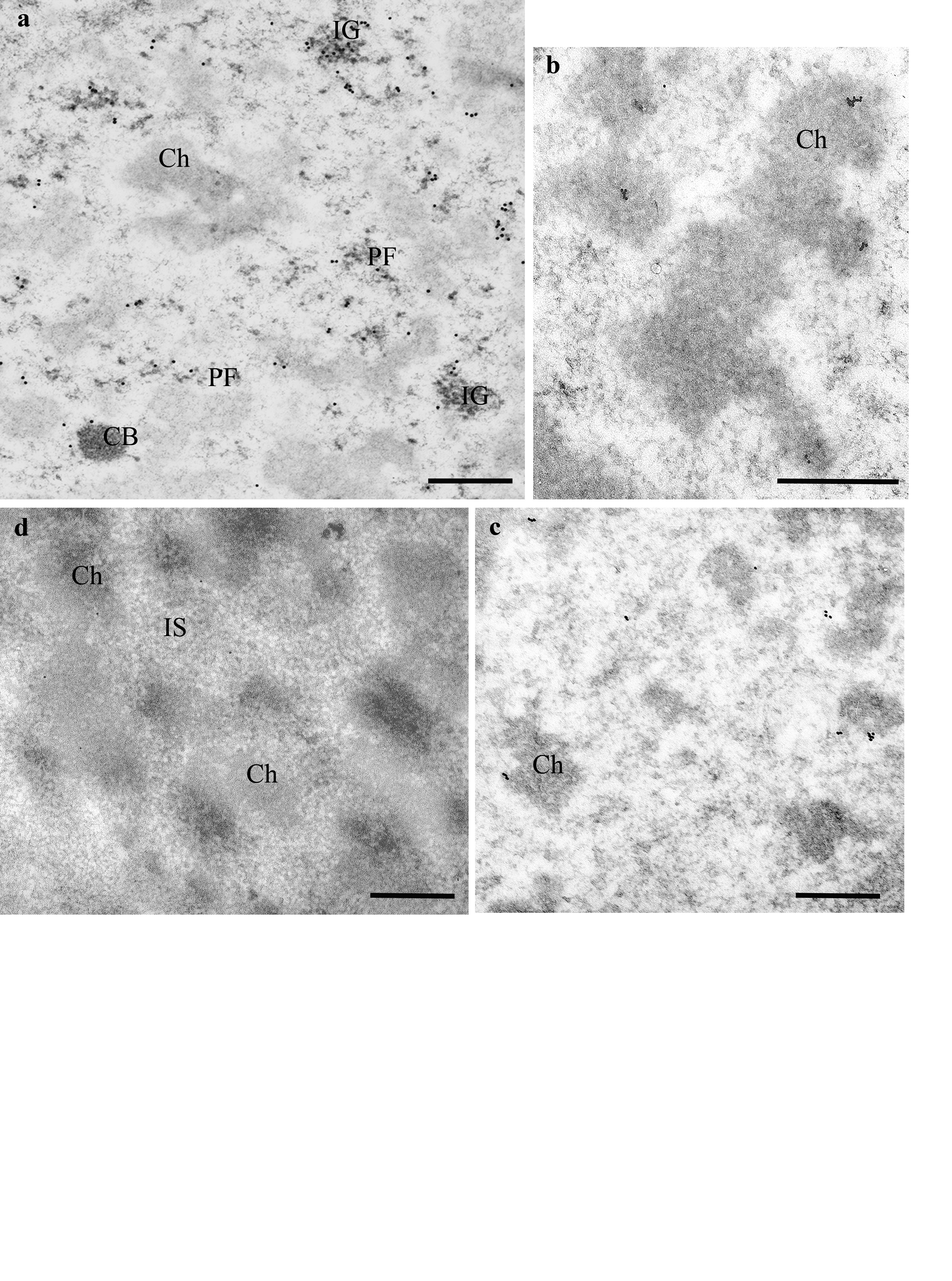

Supplement: Supplementary file 3 — Supplementary material 3 (JPEG 7.21 mb) [file 425_2012_1640_MOESM3_ESM.jpg]

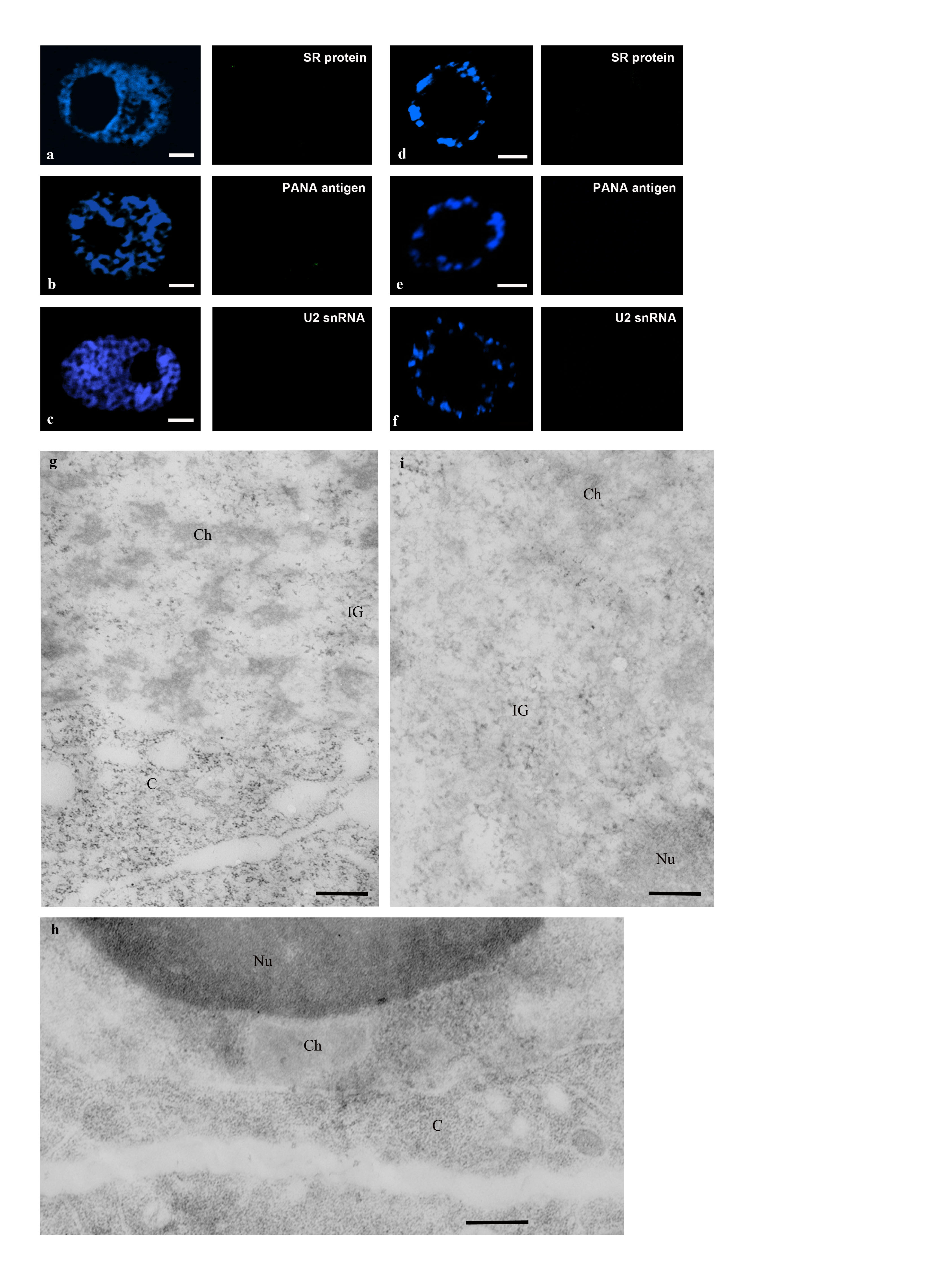

Supplement: Supplementary file 4 — Supplementary material 4 (JPEG 2.41 mb) [file 425_2012_1640_MOESM4_ESM.jpg]
